# Knowledge and awareness of genital involvement and reproductive health consequences of urogenital schistosomiasis in endemic communities in Ghana: a cross-sectional study

**DOI:** 10.1186/s12978-016-0238-5

**Published:** 2016-09-21

**Authors:** Dzidzo R. Yirenya-Tawiah, Mercy M. Ackumey, Kwabena M. Bosompem

**Affiliations:** 1 Institute of Environment and Sanitation Studies, University of Ghana, P.O Box 209, Legon, Accra, Ghana; 2 School of Public Health, University of Ghana, Legon, Accra, Ghana; 3 Noguchi Memorial Institute for Medical Research, University of Ghana, Legon, Accra, Ghana

**Keywords:** Urogenital, Schistosomiasis, Symptoms, Knowledge, Awareness, Reproductive health, Genital, Ghana

## Abstract

**Background:**

The World Health Organization, in the year 2009, renamed *Schistosomiasis haematobium* disease, urinary schistosomiasis, as urogenital schistosomiasis. This study, sought to determine whether urogenital schistosomiasis endemic community members were aware of the broadened scope of the disease and associated certain reproductive health related signs and symptoms to *S. haematobium* infection.

**Method:**

This is a cross-sectional study in which 2,585 respondents aged 15–49 years from 30 riparian communities along the lower arm of the Volta lake were interviewed using a structured questionnaire; 24 focus group discussions were also conducted. Descriptive statistics were used to determine the frequency of responses for each question posed and Chi squared tests used to determine the associations between demographic variables and variables of interest. Binary logistic regression was used to predict the probability of a reported symptom as an indicator of urogenital schistosomiasis. Thematic analysis was used to examine narratives.

**Result:**

Ninety four percent of male respondents and 88.7 % of female respondents acknowledged schistosomiasis as a water-borne disease. Only 207 out of 1,096 subjects (18.9 %) responding to questionnaire agreed to the knowledge that urogenital schistosomiasis can have reproductive health implications. A significant difference in variation in this knowledge was found between males (14.5 %) and females (7.2 %) (*p* = 0.001). The study also found that, although knowledge on HIV was high, only 12.3 % of respondents knew that urogenital schistosomiasis could facilitate the acquisition of HIV. Women who reported to have ever suffered schistosomiasis were 1.3 and 1.5 times more likely to report vaginal discharge and vaginal itch. Sexual dysfunction (11.1 %) and urethral discharge (10.6 %) were the most frequently reported symptoms among males.

**Conclusion:**

The study finds very limited knowledge on the reproductive health consequences of the disease among endemic communities. It is recommended that health education on urogenital schistosomiasis should also include issues on symptoms of the disease, reproductive health consequences and HIV transmission.

## Plain English summary

The disease urinary schistosomiasis was in 2009 renamed urogenital schistosomiasis to broaden the scope of the disease from focusing on only the urinary aspects to genital aspects of the disease. With the broadened scope in the name of the disease, this study sought to assess whether persons in endemic communities were aware of the broadened name of the disease and associated reproductive health symptoms as possible consequences of schistosomiasis.

Ninety for percent of males and almost 89 % of females knew schistosomiasis is a water-borne disease. Out of 1,096 subjects 207 (18.9 %) agreed that they knew urogenital schistosomiasis can have reproductive health implications. More male respondents had this knowledge than female respondents. Also only 12.3 % of respondents knew that urogenital schistosomiasis could facilitate the acquisition of HIV. Women who reported to have ever suffered schistosomiasis were more likely to report vaginal discharge and vaginal itch. Sexual dysfunction and urethral discharge were the most frequently reported symptoms among males.

There is limited knowledge on the reproductive health consequences of the urogenital schistosomiasis among persons living in endemic communities. It is recommended that health education on urogenital schistosomiasis should emphasize reproductive health consequences of the disease.

## Background

Urogenital schistosomiasis is one of thirteen most common chronic infections, known as neglected tropical diseases, among the world's poorest people [1, 2], Approximately 93 % of the world’s 207 million schistosomiasis infections occur in sub-Saharan Africa; 15 million cases occurring in Ghana. Two-thirds of these cases are caused by *Schistosoma haematobium*, the etiologic agent of urogenital schistosomiasis. Earlier studies on urogenital schistosomiasis, focused extensively on the urinary form of the disease commonly called urinary schistosomiasis with little attention to the genital form of the disease. The World Health Organization, in the year 2009, renamed urinary schistosomiasis as urogenital schistosomiasis to cater for the genital involvement of infection and estimated that about 45 million women of child bearing age are estimated to suffer from urogenital schistosomiasis [3]. Now that the name of the disease has been broadened to include the genital form of the disease, it is necessary for both, health professionals and endemic populations to become aware of the broadened scope of signs and symptoms to help with diagnosis and management of the infection.

A number of community based studies have highlighted the importance of urogenital schistosomiasis in reproductive and sexual health [4–8]. The tendency of the disease to have negative consequences on marriages was reported by Down et al. [5] and Friedman et al. [9].Unfortunately, urogenital schistosomiasis is mostly not perceived as a disease with serious health implications and is often accepted as part of the maturation process of life in many endemic areas [10–13]. For example, Danso-Appiah et al. [12] showed that, people who sought health care for blood in urine or blood in stool did so not for fear of suffering from schistosomiasis but rather for other suspected health problems [12]. Talaat et al. [13] also found that, in a small hamlet in Egypt although community members recognized *S. haematobium* as a health problem, they did not believe that it affected their reproductive health and were hardly aware of the possible impact of reproductive morbidity on women's arduous daily tasks [13].

Luckily, urogenital schistosomiasis is a treatable condition. Studies have shown genital lesions to regress after treatment with praziquantel [2, 14, 15]. Some protection against sexually transmitted infections (STIs) and HIV infection may also be achieved when infected individuals are treated (untreated urogenital schistosomiasis may result in various tissue lesions that can be easily infected by other bacteria and/or viruses). Unfortunately health seeking behaviour towards urinary schistosomiasis infection is generally poor. This attitude may revert if endemic community members are made aware of the reproductive health consequences of urogenital schistosomiasis infection and made to understand that the disease can be treated.

This study was therefore conducted to have an overview of what urogenital schistosomiasis endemic community members knew about the genital form of the disease. It aimed at determining endemic community members’ knowledge and awareness of the genital involvement of the disease, assess self-reported symptoms that may result from genital schistosomiasis and to determine knowledge of urogenital schistosomiasis as a risk factor to HIV transmission. For the scope of this study, reproductive health consequences is defined as any health condition that may affect a person’s reproductive system and/or ability to have satisfying sex. Authors of this paper however, acknowledge that reported signs and symptoms of urogenital schistosomiasis may also be suggestive of STIs.

## Methods

### Study area

This is a cross-sectional study conducted as part of a larger epidemiological study on female genital schistosomiasis in riparian communities on the Afram arm of the Volta Lake and the lower Volta river basin areas in Ghana.

This aspect herein, focused on soliciting subjects’ knowledge on symptoms of urogenital schistosomiasis, genital involvement of urogenital schistosomiasis disease and reproductive health. A structured question was administered and focus group discussions were held. The questionnaire captured information on demographic characteristics, knowledge about urogenital schistosomiasis, symptoms and exposure to disease. The questionnaire also solicited information on participants’ sexual activity, experience of reproductive health symptoms such as vaginal discharges and itches in the case of women and erectile dysfunction, penile itch for men and their knowledge of genital schistosomiasis as a risk factor to HIV. The inclusion criteria were: persons 15 years and above, living in study area for over a year, willingness to participate in study.

In each community, FGD participants were purposively selected after the initial screening of community members. Respondents who tested positively for urogenital schistosomiasis were serially recruited after seeking consent, A total of 205 community members participated in the FGD comprising of ten (10) male and fourteen (14) female groups with an average group size of 8 individuals within the age bracket of 18–49 years. Discussions were held with men and women separately at different times of the same day. Discussions were tape recorded and analyzed at the end of each day’s session.

### Data analysis

Quantitative data were analyzed using the Statistical Package for Social Sciences, SPSS version 18 (SPSS Inc., Chicago, Illinois, USA). Descriptive statistics were used to determine the frequency of responses for each question posed and Chi squared tests were used to determine the associations between demographic variables and variables of interest. Binary logistic regression was used to predict the probability of reported symptoms as an indicator of urogenital schistosomiasis. All quantitative analyses were done at the 5 % level of significance. Focus Group Discussions were recorded and transcribed verbatim after each interview. Transcribed discussions were analyzed against thematic concepts. These themes were derived at a consensus among the researchers after data was transcribed. Themes were based on the research questions. Some themes were ’knowledge of urogenital schistosomiasis’, reported symptoms of urogenital schistosomiases’, ‘health problems associated with urogenital schistosomiases.

## Results

Three thousand, three hundred and one (3301) questionnaires were administered to consenting subjects. Of these 2,585 (78.3 %) participants responded; 1,295 (50.1 %) respondents were males and 1,290 (49.9 %) females. The mean age of the study subjects was 29 years. More of the women interviewed (71.0 %) were married than the men (47.9 %) (*p* < 0.01). About one quarter of these women (26.2 %) were uneducated.. Of those who enrolled in school, only 42.7 % completed primary-school education, 26.0 % completed Junior High School, 4.3 % completed Senior Secondary School (SSS) and 1.4 % completed tertiary education. Fishing, petty trading, and farming, were the main occupations engaged in by the subjects. The most frequent occupation for men was fishing and women, petty trading. Only 5.8 % of participants were unemployed. Most of the study participants (60 %) had lived in their respective communities for less than 10 years. Findings from this study are presented under the following three headings; awareness and knowledge of urogenital schistosomiasis; self-reported signs and symptoms of urogenital schistosomiasis and knowledge that the disease can cause reproductive health problems including HIV.

### Awareness and knowledge of urogenital schistosomiasis

About 99.4 % of male respondents and 88.7 % of female respondents acknowledged schistosomiasis as a water-borne disease and linked the source of the disease in their community to the Volta Lake/ river. From this study, the FGD revealed the disease was identified with local names.”vudɔdɔ dolele” in Ewe, “muɔ zimi” in Dangme and “dwonsɔ mojya” in Twi (all meaning bloody urine sickness). Regarding genital schistosomiasis, the FGD showed limited knowledge of the genital form of the disease among the study participants. The presented view summarizes the general knowledge situation on genital schistosomiasis

“*I don’t know anything about GS. However it may be possible as the penis or genitalia is involved”*. (Male respondents, Pitiku).

Responses from the structured questionnaire regarding knowledge on symptoms of urogenital schistosomiasis showed a high level of awareness of blood in urine and painful urination as a symptom of the disease. Other common symptoms such as frequent passage of urine, fever and skin itch were not well known to be associated with the disease (Table 1).

**Table 1:** Knowledge of common symptoms of urogenital schistosomiasis by demographic characteristics

	Percentage of subjects with knowledge of urogenital schistosomiasis symptoms													
	Gender*		Educational level attained				Occupation*						Age group	
	(*N* = 2, 553)		(*N* = 1, 901)				(*N* = 2, 554)						(*N* = 2, 549)	
Reported knowledge of urinary schistosomiasis	Male	Female	Basic	JHS	SHS	Tertiary	Unemployed	Students	Fisher	Farmer	Trader	Others	15–24 years	>24 years
Know schistosomiasis as a disease	99.4	88.7	91.8	89.0	91.9	97.1	93.2	87.3	94.8	91.7	87.7	87.4	86.9	91.9
Symptoms														
Blood in urine Frequent urination Painful urination Fever Skin itch Don’t know	54.4 0.9 52.0 0.3 1.6 37.5	64.6 0.5 48.0 0.2 0.3 62.5	61.3 0.8 3.4 0.1 1.2 8.1	59. 2 0.6 2.9 0.2 8.3	46.8 2.7 2.7 0 0 5.4	54.3 0.0 2.9 0 0 0.0	70.5 0.0 8.0 0 0 7.5	51.9 0.5 13.3 0.5 2.3 7.9	57.2 1.1 30.7 0.0 4.2 6.1	61.6 0.9 22.7 0.5 4 9.7	63.4 0.7 24.0 0.4 2.2 11.0	70.5 0 1.3 0.0 1.3 7.7	55.5 0.6 41.3 0.4 0.3 9.8	62.0 0.8 58.7 0.2 0.7 8.1

JHS- Junior High School; SHS-Senior High School

*There was positive association using cross tabulation between knowledge and gender (*p* = 0.02) and knowledge and occupation (*p* = 0.01)

### Females reporting reproductive health symptoms

The frequency of female subjects who self-reported reproductive health symptoms are shown in Table 2. The most frequent symptom cited among female respondents was lower abdominal pain (43.3 %), followed by vaginal discharge (37.2 %) and irregular menstruation (18.7 %). The least self-reported symptom was post coital bleeding. In all 941 (72.9 %) of the respondents agreed to have suffered from at least one of the posed signs and symptoms of the disease. Vaginal discharge and vaginal itch were the only reported symptoms found to be significantly different between women who acknowledged to have suffered schistosomiasis, compared to those who never suffered schistosomiasis; (OR = 1.34, CI 1.04–1.74, *p* = 0.03), (OR = 1.48, CI = 1.05–2.09, *p*-value = 0.02) respectively (Table 3). Women who reported to have ever suffered schistosomiasis were 1.3 and 1.5 times more likely to report vaginal discharge and vaginal itch respectively, compared to women who have not suffered urogenital schistosomiasis even when adjusted for age and marital status.

**Table 2:** Female subjects reporting reproductive health symptoms

Symptom	Frequency	Percentage
Lower abdominal pain	558	43.3
Vaginal discharge	480	37.2
Vaginal itch	206	16.0
Irregular menstruation	241	18.7
Spontaneous abortion	72	5.6
Post coital bleeding	26	1.0
Infertility	21	1.6
Number of women responding to at least one reproductive health problem	941	72.9

NB. *N = 1290*

**Table 3:** Association between self-reported symptom and report of urogenital schistosomiasis among female respondents

Reproductive health condition	^a^SUS (%)	^b^SNS (%)	χ2	Crude OR	*P* value	Coefficient	Adjusted OR	CI	*P* value
Lower abdominal pain	199/448 (44.4)	359/842 (42.6)	0.38	1.01	0.97	0.05	−1.05	0.80–1.39	−0.70
Vaginal discharge	196/448 (43.8)	284/841 (33.8)	12.5	1.44	0.00	0.29	1.34	1.04–1.74	*0.03
Vaginal itch	92/448 (20.5)	114/842 (13.5)	10.6	1.50	0.02	0.39	1.48	1.05–2.09	*0.02
Irregular menstruation	90/448 (20.1 %)	151/841 (18.0)	0.88	0.91	0.65	−0.06	0.94	0.66–1.35	0.74
Miscarriage	27/448 (6.0)	45/841 (5.4)	0.25	1.1	0.64	0.24	1.27	0.74–2.18	0.39
Post coital bleeding	13/448 (2.9)	13/841 (1.5)	2.7	1.4	0.52	0.52	1.67	0.56–4.10	0.35
Infertility	10/448 (2.2)	11/841 (1.3)	1.6	0.92	0.9	0.01	1.01	0.30–3.39	0.98

^a^SU S-Subjects who reported they ever contracted urogenital schistosomiasis

Vaginal itch and discharge were adjusted for age, marital status

^b^SNS-Subjects who did not acknowledge having contracted urogenital schistosomiasis: reference category used in logistic regression model

*Vaginal discharge and itch were significant associated with urogenital schistosomiasis infection

### Self -reported symptoms of genital schistosomiasis among male respondents

Among the males, 392 of the 1295 (30.3 %) interviewed had suffered at least one of the posed reproductive health symptoms. Sexual dysfunction (9.5 %), urethral discharge (10.6 %) were the most frequently reported conditions followed by blood in semen/haemospermia (5.2 %) (Table 4). However the report on painful urination was found to be significantly different between respondents who agreed to have ever suffered urogenital schistosomiasis and those who said they had suffered the disease are 2.5 times more likely to have painful urination, (OR 2.47, CI 1.13–5.38, *p* = 0.023) (Table 5).

**Table 4:** Male subjects reporting symptoms related genital schistosomiasis symptoms

Reported Reproductive health	Frequency	Percentage
Blood in semen	67	5.2
Sexual dysfunction	123	9.5
Urethral discharge	137	10.6
Low semen volume	36	2.8
Itchy penis	11	0.8
Painful urination	37	2.9
Infertility	8	0.6
Number of men responding to any reproductive health problem	392	30.3

NB. *N* = 1295

**Table 5:** Association between self-reported symptom and report of urogenital schistosomiasis among male respondents

	Reported urinary schistosomiasis status								
Reproductive health condition	^a^SUS (%)	^b^SNS (%)	χ2	Crude OR	*P* value	Coefficient	Adjusted OR	95 % CI	*P*
Haemospermia	41/720 (5.4)	26/575 (4.5)	0.95	1.21	0.443	0.04	1.04-	0.63–1.74	0.873
Sexual dysfunction	71/716 (9.9)	52/576 (9.0)	0.31	1.15	0.459	0.10	1.11	0.76–1.63	0.598
Infertility	6/716 (0.8)	2/578 (0.3)	1.26	1.49	0.352	0.49	0.163	0.68–3.92	0.278
Urethral discharge	72/716 (10.6)	65/577 (11.3)	0.49	0.87	0.436	−0.10	0.91	0.63–1.30	0.585
Low semen volume	19/716 (2.7)	17/578 (2.9)	0.098	0.85	0.601	−0.25	0.78	0.42–1.46	0.434
Itchy penis	6/716 (0.8)	5/578 (0.9)	0.003	1.0	0.993	−0.07	0.93	0.27–3.19	0.907
Painful urination	27/716 (3.8)	10/578 (1.7)	4.5	2.24	*0.032	0.90	2.47	−1.13–5.38	*0.023

^a^SUS-Subjects who did acknowledge ever contracting urogenital schistosomiasis

^b^SNS-Subjects never contracted urogenital schistosomiasis: reference category, *Painful urination was significantly associated with urogenital schistosomiasis infection

From FGD narratives, male respondents mentioned acute abdominal pains, itchy scrotums and sexual dysfunctions as some of their reproductive health problems (see narratives below)

*“We have been experiencing severe abdominal pains of late,”* (Middle-aged man, Volivo)

*“My scrotum itches so badly. My friends also complain of itchy scrotums”.* (When probed further as to whether he had ever had the disease, he replied) *“I used to urinate blood some years ago. It has been cured with pills.”* (Male respondent Fosu)

*“Our erections are painful, and therefore we are not able to enjoy sex. We are not able to sustain an erection for long, during sexual intercourse “. (Male respondents, Kwabena Kwao)*

### Knowledge that urogenital schistosomiasis has reproductive health consequences

A total of 1, 906 responses were analysed for the question on knowledge that urogenital schistosomiasis can cause reproductive health problems. Out of these only 207 (10.9 %) agreed to this knowledge (Table 6). The study also revealed a significant difference in knowledge between males (14.5 %) and females (7.2 %) (*p* = 0.001), and knowledge of respondents engaged in different occupations (*p* = 0.001). Fishermen, reported the highest frequency with respect to knowledge that urogenital schistosomiasis could cause reproductive health problems, followed by those in school and farmers.

**Table 6:** Knowledge that urogenital schistosomiasis has reproductive health consequences

Variables	Number of Responses	Number with knowledge	(%)	(*p* value)
Respondents	1906	207	18.9	
Gender				
Male Female	949 957	138 69	14.5 7.2	*0.001
^#^Age group				
15–19 20–29 30–39 40–49 45–49	260 604 485 342 29	27 79 42 31 3	10.4 13.1 8.7 9.1 10.3	0.4
^#^Educational				
Level Basic JSS SSS Tertiary	841 485 85 27	100 49 12 3	11.9 10.1 14.1 11.1	0.9
Occupation				
Schooling Fishing Farming Trading Others Unemployed	280 403 329 620 175 99	30 34 34 37 25 7	10.7 18.4 10.3 6.0 14.3 7.1	*0.001

JHS-Junior high school; SHS-Senior high school; N=1906 –these are study participant the responded to the question posed; *p value < 0.05; ^#^Not all respondents to the question posed provided their age and attended school

### Knowledge that urogenital schistosomiasis can facilitate HIV infections

Majority of respondents (96.8 %) had knowledge of HIV as a public health problem and there was no significant difference between male and female respondents (Table 7). However, only 12.3 % of respondents knew that urogenital schistosomiasis could facilitate the acquisition of HIV.

**Table 7:** Knowledge that urogenital schistosomiasis facilitates HIV infection

	Frequency of respondents (%)			χ2 (*p* value)
	Male	Female	Total	
Aware of HIV				
Yes	1219 (97.2)	1209 (96.3)	2428 (96.8)	1.5 (0.45)
No	28 (2.2)	36 (2.9)	64 (2.6)	
Don’t know	7 (0.6)	10 (0.8)	17 (0.6)	
Aware schistosomiasis is a risk factor toHIV				
Yes	187 (15.6)	120 (9.6)	307 (12.3)	43.1 *(0.001)
No	254 (20.4)	173 (13.8)	427 (17.1)	
Don’t know	806 (64.6)	958 (76.6)	1764 (70.6)	

*p value < 0.05

Respondents who stated the likelihood of HIV infection due to urogenital schistosomiasis infection based their assertion on the fact that schistosomiasis is blood-related, and causes lacerations in the genitalia (evidenced by blood in the urine) thereby making one susceptible to HIV infection. The narrative presented summarizes the general view of respondents.

*“I don’t know if schistosomiasis can cause HIV, but if you have cuts on your vagina it is possible to get HIV after sexual intercourse. After all, we have had this disease (referring to urogenital schistosomiasis) since our grandfathers’ generations”*. (Female respondent, Dzidzorkofe)

## Discussion

The study assessed urogenital schistosomiasis endemic community members’ knowledge and awareness of reproductive health implication of urogenital schistosomiasis. In the study area, we found the prevalence of urogenital schistosomiasis among the adult population to be 46.5 % [16]. Urogenital schistosomiasis in the study area is generally considered as a health problem but with limited consequences [16]. This study on the other hand highlights the inadequate knowledge that urogenital schistosomiasis has reproductive health consequences. Only 18.9 % of respondents agreed that urogenital schistosomiasis could cause reproductive health illnesses. This finding is consistent with findings made by Talaat et al. [13] in Egypt where they found endemic community members acknowledging urogenital schistosomiasis as a disease but did not know it had reproductive health consequences [13]. This situation may be attributed to the fact that the reproductive health consequences of urogenital schistosomiasis have not had much prominence in literature and public health practice. Many of the health education interventions towards controlling urogenital schistosomiasis in Ghana do not include the reproductive health consequences of the disease. Thus very limited information on this aspect of the disease is available to endemic communities and the general public as a whole. It is generally perceived that people’s perceptions about disease risks, transmission and health consequences influences their attitudes and health seeking actions and behaviours towards the diseases [17] and consequently control strategies employed. Unfortunately, many vector-borne disease control programs have not laid emphasis on risk perception in control strategies that are employed [18]. Mostly, these programmes focus on parasite and/or vector control and health education strategies that don’t emphasize risk perception, target population’s knowledge, beliefs and behavior in the transmission and control of disease [18].

In a parallel study, we found that community members of riparian communities in the Volta Basin did not perceive urogenital schistosomiasis as an important disease and therefore either resorted to self-medication or no treatment for disease [16].Although this study found a low level of knowledge for reproductive health implications for urogenital schistosomiasis, we noted that out of those who had this knowledge, more of them were males (14.5 %) compared to female respondents (7.2 %). This variation observed in knowledge may be as a result of their experience with symptoms of urogenital diseases. The differentiation of symptoms between urinary disease and reproductive health disease may not be easily distinguishable in the case of males compared to females mainly because of the dual function of the male external genitalia (penis) in reproduction and excretion vis a vis the females that have a separate opening for the urethra and the reproductive system (vagina). For example, the signs and symptoms of an enlarged prostate include difficulty urinating, and gross or microscopic bleeding. Similar signs and symptoms may also be experienced in the case of urinary schistosomiasis. The study also noted sexual dysfunction was commonly reported by men and the manner in which they expressed this problem and others such as painful and un-sustained erection, hernia and itchy scrotum was also of particular concern, thus raising the need for studies on male urogenital schistosomiasis the area. Earlier studies had reported sexual dysfunction and scrotal involvement (although rare) in urogenital schistosomiasis infection in males [18–21]. Lopes et al. [18] also reported the case of a 31-year-old man with a 2 cm nodule in the right testis that occurred as a result of urogenital schistosomiasis infection [18].

In this study, the report of vaginal discharge and itch was found to be associated with women who reported they had ever suffered from urogenital schistosomiasis. This observations are consistent with findings by Kjetland et al. [11] who reported genital itch, malodorous and abnormally coloured discharge were significant symptoms associated with FGS infection in rural Zimbabwean women [11]. Down et al. [5] also found pelvic pain, vaginal discharge and irregular menstruation as predictors of FGS [5].

Although this survey captured only self-reported symptom data, the report of these symptoms in urogenital schistosomiasis-exposed populations may be important indicators of infection, especially where haematuria excretion among adults is often very low. We note that classic symptoms ascribed to urogenital schistosomiasis do not include vaginal discharge and itch in women.

The study also revealed HIV to be widely known as a public health problem in study area and this is confirmed by the over 90 % of respondents with this knowledge. However, knowledge that HIV infection could be facilitated by urogenital schistosomiasis was limited. Only 12 % of respondents agreed to this. Majority of participants did not know urogenital schistosomiasis as a risk factor to HIV.

Notwithstanding the relevant findings from our study, we acknowledge some limitations: Firstly, recall bias- the study was based on subjects’ recall of previous report of the disease and prevailing symptom at the time of infection 2. Subjectivity of responses-there was no inbuilt system in data gathering instrument to confirm subjects responses thus, all data gathered was based on respondents report and 3. Unavailable STI data in the study area- a major limitation to this study is unavailability of STI data in the study area to inform better the interaction between STI and UGS.

4. Data collection was not extended to investigate other confounding factors such as sexually transmitted infections that may be influencing quality of data collected. In spite of these limitations, the study brings out relevant findings on the limited knowledge on urogenital schistosomiasis in the study area.

## Conclusion

The reproductive health implication of urogenital schistosomiasis has not had much prominence in literature and public health practice. As such there is very limited knowledge on the reproductive health consequences of the disease among endemic communities and the general public and even among professionals. Noting that health risk perception among other socio-demographic characteristics influence health seeking behaviour, we recommend the need to advance urogenital schistosomiasis education to include reproductive health consequences of the disease including its potential of facilitating HIV transmission. This will provide endemic communities with knowledge required to make informed decisions on their health. Further symptom related studies should also be conducted to establish other symptoms of urogenital schistosomiasis aside from the classic blood in urine symptom.
